# Effects of Neodymium-Doped Yttrium Aluminium Garnet (Nd:YAG) Laser Capsulotomy on Visual Outcomes From a Lower-Middle Income Country

**DOI:** 10.7759/cureus.17895

**Published:** 2021-09-11

**Authors:** Narain Das, Asma Shams, Beenish Khan, Jai Kumar, Saad Nasir, Nasir M Bhatti

**Affiliations:** 1 Ophthalmology, Shaheed Mohtarma Benazir Bhutto Medical College, Karachi, PAK; 2 Ophthalmology, United Medical and Dental College, Creek General Hospital, Karachi, PAK; 3 Internal Medicine, Aga Khan University Hospital, Karachi, PAK

**Keywords:** nd:yag laser, posterior capsular opacification, complications, cataract, capsulotomy

## Abstract

Objective

Neodymium-doped yttrium aluminium garnet (Nd: YAG) laser is a non-invasive and effective means to deal with posterior capsule opacification. Although it is safe, it may have some complications. The purpose of this study was to evaluate the efficacy of Nd: YAG laser capsulotomy in terms of visual outcomes.

Methodology

This retrospective study was carried out at the eye department of Shaheed Mohtarma Benazir Bhutto Medical College, Lyari and Sindh Government Lyari General Hospital, Karachi, by using a convenient sampling technique. The duration of the study was six months from 1^st^ January 2020 to 30^th^ June 2020. 50 eyes of patients older than 20 years of age of either gender with posterior capsule opacification after cataract surgery for more than 6 months of duration, capsular fibrosis, and visual distortion due to wrinkling were included in the study.

Results

Our results show that in a total of 50 patients, the mean age was 59.08±5.84 years, of which, 20 (40%) were males. Out of 50 patients, 22 right while 28 left eyes were selected for Nd: YAG laser capsulotomy. None of the patients showed elevated intraocular pressure (IOP) after the 1^st^ week. Mean IOP was 16.84±3.63 mm of Hg on the 1^st^ day and mean IOP after 1^st^ week was 12.48±2.01 mm of Hg. Iritis was observed in 5 (10.0%) patients on the 1^st^ day and 4 (8.0%) patients on the 1^st^ week. Raised IOP was observed in 10 (20.0%) cases whereas cystoid macular edema was observed in only 1 (2%) patients on the 1^st^ day and 1^st^ week after laser therapy.

Conclusion

The study predicted that Nd: Yag laser posterior capsulotomy gives excellent results in terms of visual acuity. Complications that were associated with the Nd: Yag laser capsulotomy was a rise in intraocular pressure, cystoid macular edema, iritis, and IOL pitting.

## Introduction

A cataract is defined as opacification of the lens which may cause blurred and foggy vision of the eye [1]. Cataracts grow at a slow rate and one or both eyes can be affected [2]. Posterior capsule opacification (PCO) is a very common complication arising from catarctomy and has a frequent post-operative hurdle, which subsequently causes a large number of productions of residual lens epithelial cells (LECs) against the PCO, leading to a decline in visual acuity [3]. In the United States, the prevalence of this disease is nearly about 68% in people having above 80 years of age [4].

Cataracts can be categorized in several forms such as nuclear cataracts, which develop in the mid of the lens and creates a nucleus, which can be changed into yellow or brown, while cortical cataracts are wedge-shaped and can be developed around the borders of a nucleus. Posterior capsular cataracts develop very rapidly and affect the rear side of the lens. Congenital cataracts develop at the time of birth or during the first year of life [5]. A study revealed that glaucoma and diabetes are one of the causes of secondary cataracts. It can also be caused by drugs and after trauma [6].

The patients develop experience vague colors, blurred or double vision, circles around the light, trouble in seeing bright lights and at night. These symptoms may lead to creating problems in driving, reading, or recognizing faces. Cataracts usually arise owing to age factor but trauma or exposure to radiation is also the factors involve in cataracts, it may occur at the time of birth, or after postoperative complications [7]. Further risk elements of cataracts are diabetes, smoking, and sunlight exposure for a longer period, and alcohol [8,9]. Primarily this disease comprises of a gathering of clusters of protein or yellow and brown pigment in the lens which decreases the diffusion of light to the retina at the rear side of the eye. The diagnosis is made by visual acuity testing.

There are two modes of therapy used for management. One is surgical removal and the other is Nd: YAG laser treatment. Nd: YAG laser treatments are a non-surgical, suitable, and helpful way to treat PCO [10]. 

Mostly, patients note increased floaters in the first few days after laser capsulotomy, which ultimately become less obvious as time passes. In few patients, however, the floaters remain that take a few days for vision to become clear again. Floaters are non-dangerous clumps of cells that travel around inside the vitreous (a jelly-like material that is present inside of the eye). A person may visualize floaters as dots, circles, lines, clouds, or cobwebs. These will settle down with time and become less obvious. There is no requirement of any incisions or stitches in the laser treatment so the patient can return to their normal activities. However, vision may be unclear for a few hours afterward due to dilation of the pupil of the eye, immediately after the laser treatment. 

Several technical hitches of cataract surgery include detachment of retina and endophthalmitis [11]. In both of these, an abrupt decline in the eye vision of patients is observed. In endophthalmitis patients frequently complain of pain. While a retinal detachment presents with a defect in the unilateral visual field, blurred eye vision, and sparks of light or moving shining spots [12]. PCO is also termed as after-cataract, in which the problems with glare and light dispersion recur after successful cataract surgery, usually because of thickening of the rear or posterior capsule that surrounds the implanted lens, which is pretended PCO [13].

In some studies, it is stated that currently, Nd: YAG laser posterior treatment is being used to cope up with PCO due to its high success rate which is more than 95% [14]. In laser treatment, speedy-pulsed Nd: YAG Laser treatment in the posterior capsule is used that generates a minor hole in the axis of vision. Though it is a harmless and effective procedure, the severe problem transpires in Nd: YAG laser posterior capsulotomy is: tearing and detachment of the retina, hyphaema, cystoid macular edema (CME), Iritis or uveitis, and glaucoma [15].

The purpose of our study was to evaluate the effects of Nd: YAG laser capsulotomy on visual outcomes.

## Materials and methods

This retrospective study was carried out at the eye department of Shaheed Mohtarma Benazir Bhutto medical college and Sindh Government of Lyari General Hospital, Karachi. In this study, a convenient sampling technique was used. Ethical approval to conduct the study was obtained from the Human Research Ethics Committee of Shaheed Mohtarma Benazir Bhutto medical college and the Sindh Government of Lyari General Hospital (OPT19190). The duration of the study was six months from 1st January 2020 to 30th June 2020. Informed consent was taken from the patients and guardians. A total of 50 patients were chosen for this study.

In this study, inclusion criteria comprised of patients older than 20 years of either gender having PCO after cataract surgery for more than 6 months of duration, capsular fibrosis, and visual distortion due to wrinkling. Only patients with posterior chamber intraocular lens (IOL), having a history of glaucoma or asthmatic bronchitis or any sort of intraocular surgery in both eyes other than cataract surgery, the cornea should be cleared, were excluded from the study. The patients having no pre-existing uveitis, no systemic medications along beta-blocker or prostaglandin agents were included. Patients were also excluded if age is younger than 20 years, activity in the anterior chamber, patients using glaucoma medication or having trabeculectomy, those predisposed to retinal detachment due to peripheral retinal degenerations, post-operative cases of cataract surgery, i.e., less than six months were also excluded from the study. Thorough physical and clinical examination was completed for all the patients and all the parameters were investigated for normal levels.

Data were entered and analyzed using statistical package for social sciences (SPSS) version 20 (IBM Corporation, Armonk, NY, US) and presented in the table by calculating mean, standard mean, and deviation for quantitative data and frequency and percentages for qualitative data.

## Results

Of the total of 50 patients, the mean age was 59.08±5.84 years, in which, 20(40%) were males and 30(60%) were females. Out of 50 patients, 22 Right while 28 Left eyes were selected for Nd: YAG laser capsulotomy. We observed that majority of the patients showed no change in intraocular pressure (IOP) (Table 1). The majority of the patients have achieved their baseline that is a normal IOP. A rise in IOP was observed only in few patients as compared to baseline. None of the patients have shown elevation in IOP after 1^st^ week. On the 1st day, the mean IOP was 16.84±3.63 mmHg, wherein the mean IOP after the 1^st^ week was 12.48±2.01 mmHg.

**Table 1:** Characteristics of study participants.

Gender	Male	20(40.0%)
	Female	30(60.0%)
Comorbid conditions	Hypertension	30(60.0%)
	Diabetes	22(44.0%)
	Coronary artery disease	12(24.0%)
	Family history of hypertension	23(46.0%)
	Family history of diabetes	17(34.0%)
Intraocular pressure	Pre-laser right eye	13.28±2.67
	Pre-laser left eye	13.12±2.59
	Post laser right eye	14.72±3.51
	Post laser left eye	13.0±2.59
	1^st^-day post laser	16.84±3.63
	1^st^-week post laser	12.48±2.0

Table 1 also shows that of the patients who underwent laser capsulotomy; 22(44.0%) were diabetic, 30(60.0%) had hypertension and 12(24.0%) were suffering from coronary artery disease, so additional care of the patient was mandatory because of the high rate of post-laser complications in above-mentioned diseases. Pre laser visual acuity (VA) to check the refraction of both eyes were ranging from 1/60 to 6/36, whereas 9(18.0%) patients achieved 6/12 VA, 4(8.0%) patients achieved 6/18 VA and 2(4.0%) patients achieved 6/24 VA in post-Nd: YAG laser posterior capsulotomy in right eye refraction, as shown in Table 2. On the other hand that shows left eye refraction, 10(20.0%) patients achieved 6/12 VA, 6(12.0%) patients achieved 6/9 VA and6 (12.0%) patients achieved 6/24 VA in post Nd: YAG laser posterior capsulotomy.

**Table 2:** Visual acuity before and after Nd: YAG laser capsulotomy n = sample size. Nd: YAG = neodymium-doped yttrium aluminium garnet.

Variable		Right eye		Left eye	
		Pre laser (n=50)	Post laser (n=22)	Pre laser (n=50)	Post laser (n=28)
Visual acuity	1/60	4(8.0%)	0(0.0%)	2(4.0%)	0(0.0%)
	2/60	2(4.0%)	0(0.0%)	0(0.0%)	0(0.0%)
	3/60	3(6.0%)	0(0.0%)	2(4.0%)	0(0.0%)
	4/60	0(0.0%)	0(0.0%)	1(2.0%)	0(0.0%)
	6/60	3(6.0%)	1(2.0%)	6(12.0%)	1(2.0%)
	6/6	0(0.0%)	2(4.0%)	0(0.0%)	3(6.0%)
	6/9	1(2.0%)	3(6.0%)	4(8.0%)	6(12.0%)
	6/12	8(16.0%)	9(18.0%)	11(22.0%)	10(20.0%)
	6/18	7(14.0%)	4(8.0%)	7(14.0%)	3(6.0%)
	6/24	10(20.0%)	2(4.0%)	8(16.0%)	4(8.0%)
	6/36	12(24.0%)	1(2.0%)	7(14.0%)	1(2.0%)
	Perception of light	0(0.0%)	0(0.0%)	1(2.0%)	0(0.0%)
	No perception of light	0(0.0%)	0(0.0%)	1(2.0%)	0(0.0%)

Table 3 shows the complications that were observed after the Nd: YAG laser posterior capsulotomy. Iritis was seen in 5(10.0%) patients on the 1^st^ day and 4(8.0%) patients had iritis after the 1^st^ week. Raised IOP was observed in 10(20.0%) cases. Cystoid macular edema was seen in only 1(2%) patient on the 1^st^ day and 1^st^ week after laser therapy.

**Table 3:** Complication of Nd: YAG laser capsulotomy complications (n = 50). n = sample size. Nd: YAG = neodymium-doped yttrium aluminium garnet.

Complications		Day 1 n(%)	1^st^ Week n(%)
Iritis/Uveitis	Yes	5(10.0%)	4(8.0%)
	No	45(90.0%)	46(92.0%)
Raised intraocular pressure	Yes	10(20.0%)	0(0.0%)
	No	40(80.0%)	50(100.0%)
Cystoid macular edema	Yes	1(2.0%)	1(2.0%)
	No	49(98.0%)	49(98.0%)
Retinal tear detachment	Yes	0(0.0%)	0(0.0%)
	No	50(100.0%)	50(100.0%)
Intraocular lens pitting	Yes	15(30.0%)	1(2.0%)
	No	35(70.0%)	49(98.0%)

## Discussion

Our study focused on the results of Nd: YAG Laser Capsulotomy of 50 eyes with the changes in Visual Acuity and complications related to capsulotomy. In our study, the maximum number of patients was in between 54 - 64 years of age with a mean age of59.08±5.84years. We have also observed similar outcomes in some studies with the median age of 60 years and 58.6 years, respectively [16,17].

Investigators have made efforts to evaluate the estimated time duration between the surgery of cataracts and the development of PCO. After surgery of cataract, our study shows 6 months mean period for the development of PCO which contradicts with the other studies [18,19].

In our present study, Nd: YAG laser treatment has been very effective for those eyes which were exposed to PCO to achieve a clear pupillary passage. A considerable increase was seen in VA after Nd: YAG laser capsulotomy in our study outcomes. Few other studies reported similar findings [20,21].

In our study, a temporary upsurge in IOP was observed as a most common complication, but it gradually achieved normal levels within a week. Studies also report a temporary and abrupt rise in IOP in half of the cases inconsistent with our study findings [12,22]. Our results were per the study carried out by Hassan KS. et al. reported an average rise in IOP by 6mmHg and 3.5mmHg, respectively, after the laser treatment [23]. In our study, IOP was 16.84±3.63 mmHg on 1st day that decreased to 12.48±2.0 mmHg after one week.

According to some studies, complications of Nd: YAG laser posterior capsulotomy of damage of cornea, iritis, lens pitting was in 20% of cases, whereas cystoid macular edema (CME) was in 2% of cases, while other complications were retinal detachment (RD) and endophthalmitis [24-26]. In our study, the complications of Nd: YAG laser posterior capsulotomy were; iritis, transient raised IOP, and cystoid macular edema. However, lens pitting, corneal damage, hyphaema, retinal detachment, and endophthalmitis were not observed.

These complications may not be directly associated with YAG laser treatment but they may occur owing to reasons including the practice of surgeons and the response of patients.

## Conclusions

We conclude that Nd: YAG laser capsulotomy is a comparatively harmless, non-invasive, and a quick method to treat posterior capsular opacification. It provides tremendous outcomes in terms of development in visual acuity. We also conclude that most patients achieved better visual acuity after laser therapy. The complications that are associated with the Nd: YAG laser capsulotomy were temporary upsurge in intraocular pressure, cystoid macular edema, iritis, and lens pitting.
